# Inverse Association between Dietary Intake of Selected Carotenoids and Vitamin C and Risk of Lung Cancer

**DOI:** 10.3389/fonc.2017.00023

**Published:** 2017-02-28

**Authors:** Martine Shareck, Marie-Claude Rousseau, Anita Koushik, Jack Siemiatycki, Marie-Elise Parent

**Affiliations:** ^1^Department of Social and Environmental Health Research, London School of Hygiene and Tropical Medicine, London, UK; ^2^INRS-Institut Armand-Frappier, Laval, QC, Canada; ^3^Centre de recherche du Centre hospitalier de l’Université de Montréal (CRCHUM), Montréal, QC, Canada; ^4^Département de médecine sociale et préventive, Université de Montréal, Montréal, QC, Canada

**Keywords:** antioxidant, ascorbic acid, carotenoid, case–control study, lung neoplasm, vitamin C

## Abstract

While diets rich in fruit and vegetables appear to reduce lung cancer risk, the evidence for individual carotenoid and vitamin intakes has been judged too limited to reach firm conclusions. Data from a case–control study of lung cancer (Montreal, QC, Canada, 1996–2002) were used to investigate the role of dietary intakes of β-carotene, α-carotene, β-cryptoxanthin, lutein/zeaxanthin, lycopene, and vitamin C in lung cancer risk. In-person interviews elicited dietary information from 1,105 incident cases and 1,449 population controls. Usual frequency of consumption of 49 fruits and vegetables 2 years prior to diagnosis/interview was collected. Odds ratios (ORs) and 95% confidence intervals (CIs) between intake variables and lung cancer were estimated using logistic or polytomous regression, adjusting for potential confounding factors including a detailed smoking history. ORs associated with upper versus lower tertiles of intake were 0.66 (95% CI = 0.51–0.84) for β-carotene, 0.70 (95% CI = 0.55–0.90) for α-carotene, 0.65 (95% CI = 0.51–0.84) for β-cryptoxanthin, 0.75 (95% CI = 0.59–0.95) for lycopene, and 0.74 (95% CI = 0.58–0.96) for vitamin C. ORs suggestive of a protective effect were found for elevated intakes of β-carotene, α-carotene, β-cryptoxanthin, and lycopene in male heavy smokers and of vitamin C in female heavy smokers. Selected antioxidants were also associated with a lower risk of lung cancer in female moderate smokers, and of squamous cell carcinoma, adenocarcinoma, and small cell carcinoma. These results suggest that several dietary antioxidants found in common food sources may protect against lung cancer, even among heavy smokers.

## Introduction

Lung cancer is the leading cause of cancer mortality worldwide. Global statistics show that in 2012 alone, lung cancer was responsible for an estimated 1.6 million deaths or 19.4% of all cancer-related deaths (1). Since survival remains low, the main hope for reducing the burden of this disease lies in prevention. Cigarette smoking is the foremost risk factor for lung cancer, accounting for up to 90% of all cases (2). Large-scale smoking prevention and cessation efforts have been attempted (3–5), and efforts in that direction must be pursued. However, other modifiable risk factors must be identified so that all possible lung cancer prevention strategies can be implemented. The recognized multifactorial etiology of lung cancer suggests that factors other than smoking, such as diet, can influence its occurrence (6).

Results from a large systematic review published by the World Cancer Research Fund (WCRF) in 2007, as well as the 2016 update of this report, suggested a protective role of diets rich in fruit and vegetables (7, 8). These foods are thought to protect against lung cancer due to their content in micronutrients that have chemopreventive properties such as antioxidant activity. Antioxidants found in the highest concentrations in both the human diet and serum samples include vitamin C and the specific carotenoids β-carotene, α-carotene, β-cryptoxanthin, lutein, zeaxanthin, and lycopene (9). In its assessment, the WCRF stated that while the evidence suggests that foods rich in carotenoids probably decrease lung cancer risk, no firm conclusion could yet be reached about the role of individual carotenoids or vitamin C as the evidence for these was too limited or inconsistent, respectively (7).

Although several studies have examined associations between individual carotenoids and vitamin C and lung cancer, including case–control studies (10–17), cohort studies (18–31), pooled analyses (32, 33), and meta-analyses (34–36), results remain equivocal. This may be attributable to a combination of small sample sizes in some studies (10, 12–19, 21–24, 28) and inadequate control of smoking in others, usually because they were not able to control for time since quitting in addition to other dimensions of smoking (25, 28, 30). Most studies focused exclusively on one sex or the other (11, 12, 14–19, 21, 23, 24, 26–28, 31), and if there was effect modification by sex, this would lead to heterogeneity of results. A few studies have suggested that antioxidants may relate differently to lung cancer risk depending on sex (10, 20, 25), smoking intensity (20, 22, 27, 31, 37) and histological subtype of the tumor (13, 16, 26, 36), and heterogeneity of risk among such subgroups may have contributed to inconsistencies in the literature.

This prompted us to examine the relationship between intake of selected antioxidants and the risk of lung cancer in a large population-based case–control study, using updated food composition databases and detailed control for confounding by several different dimensions of smoking. We examined potential effect modification by sex and by smoking intensity on the antioxidant–lung cancer relationship, and examined the association between these micronutrients and four tumor histological subtypes.

## Materials and Methods

### Study Population and Data Collection

Data from a population-based case–control study conducted in Montreal, Québec (QC), Canada, were used. Eligible subjects were men and women, aged 35–75 years and living in the Montreal area. Cases were ascertained from 18 hospitals in the Montreal Metropolitan region, providing almost complete coverage of lung cancer diagnoses in the area (~98%). Potential cases were identified through active monitoring of hospital pathological reports and included histologically confirmed primary lung cancer cases diagnosed between January 1996 and December 1997. Concurrently, controls were randomly selected from the general population through electoral lists. In Canada, these lists include the names and addresses of practically all Canadian citizens residing in the country and are updated through active population enumeration. Controls were frequency matched to cases by age (5-year group), sex, and electoral district (comprising some 40,000 electors). The study and protocol were approved by the following Research Ethics Committees: the Institut National de la Recherche Scientifique, Hôpital Notre-Dame, Hôpital St-Luc, Hôtel-Dieu de Montréal, Hôpital Maisonneuve-Rosemont, Hôpital Jean-Talon, Hôpital Charles-Lemoyne, Hôpital Fleury, Hôpital du Sacré-Coeur de Montréal, Hôpital Lachine, Hôpital Santa Cabrini, Jewish General Hospital, Royal-Victoria Hospital, Montreal General Hospital, St-Mary’s Hospital, Lakeshore General Hospital, Hôpital de Lasalle, Montreal Chest Hospital, Hôpital de Verdun, and Hôpital Pierre-Boucher. The study was carried out in accordance with their recommendations. All subjects provided written informed consent in accordance with the Declaration of Helsinki. A total of 1,434 cases and 2,182 controls were invited to participate in the study: 1,203 (83.9%) cases and 1,513 (70.6%) controls accepted. Trained interviewers conducted in-person interviews with the subject or a proxy respondent (if the subject was deceased or too ill to respond). Information was collected on a wide range of factors including subjects’ socioeconomic background, detailed occupational history, smoking history (smoking status, changes in smoking intensity levels and interruptions, cigarette-years, time since cessation), lifetime intake of alcoholic beverages (wine, beer, spirits), and dietary intake.

### Dietary Intake

Diet was assessed using a semiquantitative food frequency questionnaire (FFQ) adapted from the instrument developed by the Canadian Cancer Registries Epidemiology Research Group (38), which was based on two extensively validated questionnaires: the National Cancer Institute’s Block Questionnaire (39) and the Nurses’ Health Study FFQ (40). Modifications were made to reflect the diet of the study population and to capture major dietary sources of carotenoids and vitamin C among adults living in Québec, Canada. The FFQ was pre-tested for face validity in a subgroup of the target population to ensure that questions were well understood. The questionnaire covered 77 food items, including 49 fruits and vegetables grouped into 25 individual statements. Frequency of intake 2 years prior to diagnosis or interview, in terms of a typical portion size specified for each question, was reported as “7 or more times per week,” “4 to 6 times per week,” “1 to 3 times per week,” “1 to 3 times per month,” and “never or less than once per month.” The nutrient content of foods was extracted from the Canadian Nutrient File (version 2007b) (41). For categories with closed frequency ranges, the mid-point value was used to assign a weekly frequency of intake of each food (42, 43). The following values were assigned to each category: 7, 5, 2, 0.5, or 0 times per week. Individual daily intakes of β-carotene, α-carotene, β-cryptoxanthin, lutein/zeaxanthin, lycopene, and vitamin C were then calculated by multiplying the weekly frequency of intake of each food by the nutrient content of the specified portion size and then summing the contributions from all foods and dividing by 7. The food sources of each nutrient are presented in Table S1 in Supplementary Material. Nutrient intakes were adjusted for total energy with the residual method using the predicted nutrient intake for the median daily energy intake among women and men in the sample. Adjusting nutrient intakes for energy reduces measurement error for specific nutrients and removes extraneous variation, allowing the direct evaluation of variation due to dietary composition rather than absolute nutrient intakes (42). Energy-adjusted nutrient values were categorized into tertile levels of intake based on the frequency distribution among controls. In all, 162 subjects (6%) were excluded from the dietary analyses either because they had not filled in the dietary section or because they had answered less than 50% of the dietary questions. It has indeed been observed previously that it is reasonable to exclude subjects for whom 50% or more of items on a FFQ are left unanswered, if missing values are randomly distributed across the questionnaire (44). The final sample for analysis thus consisted of 2,554 subjects, i.e., 1,105 lung cancer cases and 1,449 population controls.

### Statistical Analyses

Logistic regression models were used to estimate the risk of lung cancer associated with micronutrient intakes. Adjusted odds ratios (ORs) and 95% confidence intervals (CIs) were estimated comparing second and third tertile levels of intake (referred to as medium and high intakes) to the first tertile level of intake (low intake) for each micronutrient. Polytomous regression models were applied to estimate the association between micronutrient intakes and risk of squamous cell carcinoma, adenocarcinoma, small cell carcinoma, and large cell carcinoma. Covariates included in regression models were age (continuous), sex (woman or man), respondent status (self or proxy), ethnic origin (French ancestry, English/Irish/Scottish ancestry, other), education level (elementary, secondary, post-secondary), body mass index (BMI) 2 years prior to interview (continuous), total daily energy intake (in kilocalories, continuous), and cigarette smoking. Cigarette smoking was modeled using three variables as suggested by Leffondré et al. (45), i.e., (i) ever smoked (yes or no); (ii) natural logarithm of cigarette-years (number of cigarettes smoked per day multiplied by smoking duration in years, continuous); and (iii) time since cessation (in years, continuous). Potential confounding by other variables, including income, cumulative intake of alcoholic beverages, and exposure to asbestos at work (46), was evaluated by a 10% change-in-estimate criterion, and none of those covariates were empirical confounders in this dataset. Effect modification by sex and by lifetime smoking intensity was examined by including relevant cross-products in the multivariate models. Tests for linear trend across intake tertiles were carried out by modeling nutrient intake tertiles as continuous variables. Statistical analyses were performed using SPSS version 19.0 (47).

## Results

### Sample Description

A description of study participants is provided in Table 1. Cases and controls were similar with respect to age but differed significantly on sociodemographic characteristics, their reliance on proxy respondents, BMI, history of cigarette smoking, and lifetime alcohol intake. As compared to controls, cases were more likely to be of French ancestry and to be less educated. Proxy respondents provided information for a significantly larger proportion of cases than controls. Compared to controls, cases were more likely to have ever smoked and to have greater values for cigarette-years, and were less likely to be former smokers. The distribution of histological subtypes differed by sex. Among males, a similar proportion of cases had been diagnosed with squamous cell carcinoma and adenocarcinoma, while adenocarcinoma was the most prevalent tumor histology among women.

**Table 1:** **Selected characteristics of cases and population controls, by sex, Montreal, QC, Canada, 1996–2002**.

Characteristic	Men		Women	
	Cases (*n* = 690)	Controls (*n* = 870)	Cases (*n* = 415)	Controls (*n* = 579)
Mean age (SD)	64.3 (7.8)	65.0 (7.6)	61.4 (9.4)	61.5 (9.3)
Ethnic origin (%)*				
French	78.3	64.5	79.5	69.1
English, Irish, or Scottish	4.5	6.2	8.4	4.0
Other	17.2	29.3	12.0	26.9
Education level (%)*				
Elementary	48.3	35.3	35.2	26.3
Secondary	39.6	41.6	52.0	44.0
Post-secondary	12.2	23.1	12.8	29.7
Mean income, $ (SD)*	33,130 (14,979)	35,244 (14,237)	33,997 (16,048)	38,641 (14,708)
Self-respondent (%)*	61.0	90.2	67.7	95.3
**Histology (%)**				
Small cell carcinoma	17.0	NA	16.6	NA
Squamous cell carcinoma	35.9	NA	19.0	NA
Adenocarcinoma	32.5	NA	48.7	NA
Large cell carcinoma	9.6	NA	8.9	NA
Other	5.0	NA	6.8	NA
Smoking status (%)*				
Never smokers	22.5	62.1	18.1	69.8
Former smokers	10.1	9.3	8.7	8.3
Current smokers	67.4	28.6	73.3	21.9
Mean cigarette-years^a^ (SD)*	1,539.2 (896.5)	824.8 (783.6)	989.0 (586.0)	290.3 (456.4)
Mean years since quitting smoking^b^ (SD)*	4.5 (8.0)	11.2 (13.0)	2.5 (5.7)	4.9 (10.0)
Mean BMI (SD) (kg/m^2^)*	25.1 (4.2)	26.0 (3.7)	24.3 (5.0)	25.4 (4.5)
Ever exposure to asbestos (%)	18.7	17.0	0	0.7
Mean cup-years of alcohol (SD)*	107.4 (212.4)	84.6 (204.7)	17.4 (47.0)	9.9 (47.3)

*BMI, body mass index; NA, not applicable*.

*^a^Among ever smokers*.

*^b^Among quitters*.

*Characteristics on which cases and controls differed at P = 0.05 significance level.

Median intake values of selected antioxidants among cases and controls are presented in Table 2. Among both men and women, and for all antioxidants studied, cases had lower median daily intakes than controls.

**Table 2:** **Median micronutrient intakes^a^ and interquartile ranges for cases and population controls, by sex, Montreal, QC, Canada, 1996–2002**.

Median micronutrient intakes	Men		Women	
	Cases (*n* = 690)	Controls (*n* = 870)	Cases (*n* = 415)	Controls (*n* = 579)
β-Carotene (μg/day) (IQR)	3,810 (4,883)	5,243 (5,225)	3,253 (3,043)	5,205 (4,196)
α-Carotene (μg/day) (IQR)	1,185 (1,566)	1,702 (1,610)	1,096 (1,084)	1,384 (1,508)
β-Cryptoxanthin (μg/day) (IQR)	86 (128)	140 (161)	76 (93)	127 (143)
Lutein/zeaxanthin (μg/day) (IQR)	1,164 (2,378)	1,784 (3,617)	1,053 (1,409)	1,842 (3,001)
Lycopene (μg/day) (IQR)	15,888 (10,878)	16,969 (9,285)	11,911 (11,902)	16,175 (10,958)
Vitamin C (mg/day) (IQR)	70 (91)	104 (99)	61 (71)	105 (74)

*IQR, interquartile range*.

*^a^Micronutrient intakes are unadjusted for energy to facilitate comparison with general population intakes*.

### Correlation between Individual Micronutrients

As expected, most individual micronutrients were highly correlated with one another (Table S2 in Supplementary Material). One notable exception was lycopene, which showed correlation coefficients with other micronutrients ranging from 0.34 to 0.50, whereas the correlation between the other micronutrients ranged from 0.52 to 0.94. This high level of collinearity impeded our ability to estimate the independent effect of each micronutrient on lung cancer risk, and this should be kept in mind when interpreting the results.

### Main Effects

Table 3 presents adjusted ORs for lung cancer associated with medium and high, versus low intake levels of energy-adjusted micronutrients. Sex did not emerge as an effect modifier of the association between micronutrients and lung cancer, with *P* values for the interaction terms ranging from 0.389 for α-carotene to 0.925 for β-cryptoxanthin in the fully adjusted models; therefore, results based on the entire sample are presented, after adjustment for sex. When compared to those with low levels of intake, subjects in the highest intake level of β-carotene, α-carotene, β-cryptoxanthin, lycopene, and vitamin C had a statistically significant lower risk of lung cancer. For all these micronutrients except vitamin C, significant dose–response trends were observed.

**Table 3:** **Adjusted ORs and tests for trend for lung cancer risk according to dietary intakes of specific carotenoids and vitamin C in tertiles, Montreal, QC, Canada, 1996–2002**.

Energy-adjusted micronutrient tertile^a^	*n*_ca_	*n*_co_	OR^b^	95% CI
**β-Carotene (μg/day)**				
T1: <4,015	474	483	(ref.)	
T2: 4,015–6,515	393	483	0.83	0.66, 1.06
T3: >6,515	238	483	**0.66**	**0.51, 0.84**
*P* for trend^c^			**<0.001**	
**α-Carotene (μg/day)**				
T1: <1,131	407	483	(ref.)	
T2: 1,131–2,015	437	483	1.01	0.80, 1.28
T3: >2,015	261	483	**0.70**	**0.55, 0.90**
*P* for trend			**0.01**	
**β-Cryptoxanthin (μg/day)**				
T1: <100	504	483	(ref.)	
T2: 100–175	398	483	0.88	0.70, 1.01
T3: >175	203	483	**0.65**	**0.51, 0.84**
*P* for trend			**<0.001**	
**Lutein/zeaxanthin (μg/day)**				
T1: <1,249	407	483	(ref.)	
T2: 1,249–2,827	445	483	1.17	0.91, 1.51
T3: >2,827	253	483	1.01	0.78, 1.31
*P* for trend			0.38	
**Lycopene (μg/day)**				
T1: <12,425	431	483	(ref.)	
T2: 12,425–18,401	347	483	0.79	0.62, 1.10
T3: >18,401	327	483	**0.75**	**0.59, 0.95**
*P* for trend			**0.03**	
**Vitamin C (mg/day)**				
T1: <81	525	483	(ref.)	
T2: 81–122	355	483	0.81	0.64, 1.02
T3: >122	225	483	**0.74**	**0.54, 0.96**
*P* for trend			0.16	

*CI, confidence interval; *n*_ca_, number of exposed cases; *n*_co_, number of exposed controls; OR, odds ratio; (ref.), reference category*.

*^a^Tertiles based on the frequency distribution of energy-adjusted nutrient intakes among controls (men and women combined)*.

*^b^Adjusted for age (continuous), sex (man or woman), respondent status (self or proxy), ethnic background (French ancestry, English/Irish/Scottish ancestry, other), education (primary, secondary, post-secondary), ever smoked (yes or no), natural log of cigarette-years (continuous), years since quitting smoking (continuous), energy intake (continuous), and body mass index (continuous)*.

*^c^Tests for trend were carried out by modeling nutrient intake tertiles as continuous variables*.

*Bold font indicates P < 0.05*.

### Associations by Cumulative Smoking Intensity

Tables 4 and 5 show associations between micronutrient intakes and lung cancer risk by cumulative smoking intensity, in men and women, respectively. Results are presented separately for men and women since cumulative smoking intensity varied substantially between sexes, and the sample included a substantial group of never smoking women for whom potential confounding by smoking would not be an issue. As well, although most nutrient-smoking intensity interaction terms did not reach statistical significance, with *P* values in the fully adjusted models ranging between 0.046 for β-cryptoxanthin in women and 0.910 for vitamin C in men, stratified results allow for comparison with recent studies (31). As very few men had never smoked, never and light smokers were combined into a single category. In almost all strata, the point estimates were lower in the high micronutrient category than in the low micronutrient category, although not all of these were statistically significantly lower. In general, the strongest protective effects in men were observed among heavy intensity smokers, whereas in women, they occurred among intermediate intensity smokers. Among men who were never and light smokers, the only statistically significant association was observed among those in the second intake level of lutein/zeaxanthin, who were at greater risk of lung cancer compared to those in the lowest intake level. There were no significant associations among men who smoked at intermediate intensity levels. Heavy smoking men were at lesser risk of lung cancer when consuming high intakes of all micronutrients, although a statistically significant inverse association was found only for β-carotene, α-carotene, β-cryptoxanthin, and lycopene. There were no significant associations among women who never smoked, although it should be noted that numbers of subjects were small. Among women in the intermediate smoking intensity category, intakes in the third tertile of β-carotene and second tertiles of β-cryptoxanthin, lycopene, and vitamin C were associated with a lower lung cancer risk, while both medium and high intakes of vitamin C showed an inverse relationship in heavy smoking women. Two-sided tests for trend assessing the dose–response relationship were significant at the 0.05 level for β-carotene, α-carotene, β-cryptoxanthin, and lycopene in heavy smoking men, as well as for vitamin C in heavy smoking women.

**Table 4:** **Adjusted ORs and tests for trend for lung cancer risk according to dietary intakes of specific carotenoids and vitamin C in tertiles^a^ in men, stratified by cumulative smoking intensity, Montreal, QC, Canada, 1996–2002**.

Energy-adjusted micronutrient tertile	Men									
	Never/light intensity smokers^b,c^ ≤700.5 cig-yrs			Intermediate intensity smokers^c^ 705–1,305 cig-yrs			Heavy intensity smokers^c^ ≥1,310 cig-yrs			
	*n*_ca_	OR^c^	95% CI	*n*_ca_	OR	95% CI	*n*_ca_	OR	95% CI	*P* for interaction
**β-Carotene (μg/day)**										0.692
T1: <4,015	28	(ref.)		110	(ref.)		159	(ref.)		
T2: 4,015–6,760	29	0.78	0.41, 1.46	109	1.00	0.63, 1.60	116	0.84	0.51, 1.36	
T3: >6,760	26	0.78	0.42, 1.48	54	0.78	0.47, 1.31	59	**0.49**	**0.28, 0.83**	
*P* for trend^d^		0.45			0.39			**0.01**		
**α-Carotene (μg/day)**										0.282
T1: <1,177	24	(ref.)		102	(ref.)		140	(ref.)		
T2: 1,177–2,064	32	0.85	0.45, 1.61	112	1.20	0.75, 1.92	124	1.02	0.61, 1.68	
T3: >2,064	27	1.06	0.56, 2.02	59	0.81	0.49, 1.34	70	**0.53**	**0.31, 0.89**	
*P* for trend		0.85			0.48			**0.02**		
**β-Cryptoxanthin (μg/day)**										0.569
T1: <103	31	(ref.)		125	(ref.)		176	(ref.)		
T2: 103–178	33	1.07	0.59, 1.93	95	0.70	0.44, 1.11	106	0.92	0.57, 1.50	
T3: >178	19	0.67	0.34, 1.31	53	0.85	0.50, 1.45	52	**0.58**	**0.34, 0.98**	
*P* for trend		0.27			0.41			0.06		
**Lutein/zeaxanthin (μg/day)**										0.197
T1: <1,249	14	(ref.)		101	(ref.)		145	(ref.)		
T2: 1,249–2,875	38	**2.24**	**1.01, 4.54**	107	0.94	0.57, 1.58	123	1.25	0.74, 2.11	
T3: >2,875	31	1.94	0.92, 4.12	65	0.95	0.56, 1.61	66	0.87	0.50, 1.52	
*P* for trend		0.12			0.85			0.79		
**Lycopene (μg/day)**										0.063
T1: <13,080	30	(ref.)		98	(ref.)		139	(ref.)		
T2: 13,080–19,281	23	0.77	0.40, 1.47	90	0.92	0.58, 1.48	103	0.75	0.44, 1.25	
T3: >19,281	30	1.10	0.59, 2.04	85	1.09	0.68, 1.77	92	**0.48**	**0.29, 0.79**	
*P* for trend		0.76			0.73			**<0.01**		
**Vitamin C (mg/day)**										0.910
T1: <83	31	(ref.)		128	(ref.)		177	(ref.)		
T2: 83–129	28	0.73	0.39, 1.37	100	1.00	0.64, 1.57	106	0.88	0.55, 1.40	
T3: >129	24	0.60	0.31, 1.14	45	0.90	0.52, 1.54	51	0.69	0.39, 1.21	
*P* for trend		0.12			0.73			0.20		

*CI, confidence interval; cig-yrs, cigarette-years; *n*_ca_, number of exposed cases; (ref.), reference category*.

*^a^Tertiles were based on the frequency distribution of energy-adjusted micronutrient intakes among controls (men only)*.

*^b^167 were never smokers (16 cases and 151 controls)*.

*^c^Adjusted for age (continuous), respondent status (self or proxy), ethnic background (French ancestry, English/Irish/Scottish ancestry, other), education (primary, secondary, post-secondary), energy intake (continuous), body mass index (continuous), years since quitting smoking (continuous), and cigarette-years (continuous)*.

*^d^Tests for trend were carried out by modeling nutrient intake tertiles as continuous variables*.

*Bold font indicates P < 0.05*.

**Table 5:** **Adjusted ORs and test for trend for lung cancer risk according to dietary intakes of specific carotenoids and vitamin C in tertiles^a^ in women, stratified by cumulative smoking intensity, Montreal, QC, Canada, 1996–2002**.

Energy-adjusted micronutrient tertile	Women									
	Never smokers^b^ 0 cig-yrs			Intermediate intensity smokers^c^ 1–850 cig-yrs			Heavy intensity smokers^c^ ≥ 850.5 cig-yrs			
	*n*_ca_	OR	95% CI	*n*_ca_	OR	95% CI	*n*_ca_	OR	95% CI	*P* for interaction
**β-Carotene (μg/day)**										0. 765
T1: <4,017	11	(ref.)		52	(ref.)		114	(ref.)		
T2: 4,017–5,978	11	0.87	0.34, 2.22	49	0.88	0.46 1.67	80	0.71	0.35, 1.46	
T3: >5,978	10	0.68	0.26, 1.79	27	**0.51**	**0.26, 0.98**	61	0.79	**0.36, 1.73**	
*P* for trend^d^		0.44			0.05			0.50		
**α-Carotene (μg/day)**										
T1: <1,073	10	(ref.)		43	(ref.)		87	(ref.)		0. 396
T2: 1,073–1,960	13	0.83	0.32, 2.16	55	0.97	0.50, 1.90	114	1.95	0.92, 4.14	
T3: >1,960	9	0.76	0.28, 2.07	30	0.51	0.26, 1.01	54	0.83	0.39, 1.75	
*P* for trend		0.59			0.05			0.76		
**β-Cryptoxanthin (μg/day)**										**0.046**
T1: <99	9	(ref.)		56	(ref.)		115	(ref.)		
T2: 99–172	15	1.61	0.63, 4.13	42	**0.39**	**0.20, 0.76**	99	1.17	0.58, 2.38	
T3: >172	8	0.81	0.28, 2.32	30	0.56	0.28, 1.11	41	0.52	0.25, 1.11	
*P* for trend		0.68			0.07			0.14		
**Lutein/zeaxanthin (μg/day)**										0.564
T1: <1,255	7	(ref.)		51	(ref.)		90	(ref.)		
T2: 1,255–2,777	14	1.40	0.49, 3.98	49	0.77	0.39, 1.52	114	1.44	0.64, 3.25	
T3: >2,777	11	1.08	0.37, 3.16	28	0.76	0.37, 1.54	51	0.98	0.45, 2.14	
*P* for trend		0.96			0.43			0.96		
**Lycopene (μg/day)**										0.653
T1: <11,192	13	(ref.)		51	(ref.)		101	(ref.)		
T2: 11,192–17,003	10	0.72	0.28, 1.83	38	**0.45**	**0.23, 0.88**	76	0.92	0.45 1.88	
T3: >17,003	9	1.00	0.39, 2.62	39	0.60	0.31, 1.15	78	1.20	0.59, 2.48	
*P* for trend		0.95			0.11			0.64		
**Vitamin C (mg/day)**										0.316
T1: <79	10	(ref.)		62	(ref.)		131	(ref.)		
T2: 79–114	13	1.28	0.49, 3.32	35	**0.47**	**0.25, 0.91**	71	**0.40**	**0.19, 0.84**	
T3: >114	9	0.77	0.27, 2.15	31	0.60	0.31, 1.17	53	**0.45**	**0.21, 0.95**	
*P* for trend		0.58			0.10			**0.02**		

*CI, confidence interval; cig-yrs, cigarette-years; *n*_ca_, number of exposed cases; (ref.), reference category*.

*^a^Tertiles were based on the frequency distribution of energy-adjusted micronutrient intakes among controls (women only)*.

*^b^Adjusted for age (continuous), respondent status (self or proxy), ethnic background (French ancestry, English/Irish/Scottish ancestry, other), education (primary, secondary, post-secondary), energy intake (continuous), and body mass index (BMI) (continuous)*.

*^c^Adjusted for age (continuous), respondent status (self or proxy), ethnic background (French ancestry, English/Irish/Scottish ancestry, other), education (primary, secondary, post-secondary), energy intake (continuous), BMI (continuous), years since quitting smoking (continuous), and cigarette-years (continuous)*.

*^d^Tests for trend were carried out by modeling nutrient intake tertiles as continuous variables*.

*Bold font indicates P < 0.05*.

### Associations by Histological Subtype of the Tumor

Odds ratios for the association between micronutrient intakes and each of the four most common histological subtypes of lung cancer are presented in Table 6. There were no clear differences in the patterns of results between squamous cell carcinoma, adenocarcinoma, and small cell carcinoma. While there was some variation in the point estimates, the confidence limits for any given micronutrient overlapped considerably between the histologic subtypes. When compared to subjects in the lowest tertile level of intake, those with the highest intake levels of β-carotene, α-carotene, lycopene, and vitamin C had a statistically significant lower risk of squamous cell carcinoma, while both medium and high intakes of β-cryptoxanthin suggested a protective effect for this histological subtype. High intakes of β-carotene and α-carotene were associated with a reduced risk of adenocarcinoma, while both medium and high intakes of β-cryptoxanthin and lycopene suggested a protective effect for small cell carcinoma. High intakes of all other antioxidants were associated with a non-significant decrease in risk of squamous cell carcinoma, adenocarcinoma, and small cell carcinoma, save for lutein/zeaxanthin which was related to a slightly increased risk of small cell carcinoma. High intakes of β-carotene, β-cryptoxanthin, lutein/zeaxanthin, lycopene, and vitamin C were similarly associated with statistically non-significant increases in risk of large cell carcinoma, although it should be noted that these results were based on relatively few cases. Statistically significant trends (two-sided, *P* < 0.05) for a lower risk of squamous cell carcinoma were observed with increased levels of β-carotene, α-carotene, β-cryptoxanthin, lycopene, and vitamin C, and similarly between adenocarcinoma and β-carotene and α-carotene, as well as between small cell carcinoma and both β-cryptoxanthin and lycopene.

**Table 6:** **Adjusted ORs and tests for trend for risk of four histologic subtypes of lung cancer according to dietary intakes of specific carotenoids and vitamin C in tertiles^a^, Montreal, QC, Canada, 1996–2002**.

Energy-adjusted micronutrient tertile		Squamous cell carcinoma			Adenocarcinoma			Small cell carcinoma			Large cell carcinoma		
		*n*_ca_	OR^b^	95% CI	*n*_ca_	OR	95% CI	*n*_ca_	OR	95% CI	*n*_ca_	OR	95% CI
**β-Carotene (μg/day)**												
T1: <4,015	147	(ref.)		186	(ref.)		79	(ref.)		35	(ref.)	
T2: 4,015–6,515	116	0.76	0.55, 1.05	149	0.80	0.60, 1.07	67	0.90	0.60, 1.35	40	1.17	0.70, 1.97
T3: >6,515	64	**0.56**	**0.40, 0.80**	91	**0.62**	**0.45, 0.85**	40	0.76	0.49, 1.19	28	1.08	0.62, 1.88
*P* for trend^c^		**<0.001**			**<0.01**			0.23			0.75	
**α-Carotene (μg/day)**												
T1: <1,131	117	(ref.)		157	(ref.)		74	(ref.)		35	(ref.)	
T2: 1,131–2,015	131	0.99	0.71, 1.37	172	1.05	0.78, 1.40	66	0.80	0.53, 1.20	43	1.15	0.68, 1.92
T3: >2,015	79	**0.70**	**0.49, 0.99**	97	**0.68**	**0.50, 0.94**	46	0.70	0.45, 1.08	25	0.82	0.46, 1.44
*P* for trend		**0.05**			**0.02**			0.09			0.51	
**β-Cryptoxanthin (μg/day)**												
T1: <100	166	(ref.)		171	(ref.)		99	(ref.)		37	(ref.)	
T2: 100–175	111	**0.72**	**0.53, 0.98**	169	1.08	0.81, 1.44	57	**0.66**	**0.45, 0.99**	42	1.29	0.79, 2.13
T3: >175	50	**0.47**	**0.33, 0.69**	86	0.77	0.56, 1.06	30	**0.60**	**0.37, 0.96**	24	1.19	0.67, 2.12
*P* for trend		**<0.001**			0.17			**0.01**			0.48	
**Lutein/zeaxanthin (μg/day)**												
T1: <1,249	120	(ref.)		159	(ref.)		72	(ref.)		34	(ref.)	
T2: 1,249–2,827	145	1.30	0.92, 1.83	159	1.00	0.73, 1.37	78	1.27	0.82, 1.97	37	1.06	0.60, 1.87
T3: >2,827	62	0.91	0.62, 1.33	108	0.99	0.71, 1.37	36	1.03	0.64, 1.67	32	1.38	0.79, 2.41
*P* for trend		0.80			0.69			0.79			0.26	
**Lycopene (μg/day)**												
T1: <12,425	123	(ref.)		169	(ref.)		81	(ref.)		30	(ref.)	
T2: 12,425–18,401	104	0.79	0.57, 1.10	137	0.83	0.62, 1.11	50	**0.59**	**0.38, 0.89**	41	1.34	0.80, 2.28
T3: >18,401	100	**0.70**	**0.50, 0.98**	120	0.76	0.56, 1.03	55	**0.65**	**0.43, 0.99**	32	1.14	0.65, 1.98
*P* for trend		**0.04**			0.08			**0.04**			0.66	
**Vitamin C (mg/day)**												
T1: <80.6	161	(ref.)		198	(ref.)		97	(ref.)		40	(ref.)	
T2: 80.6–121.7	113	0.81	0.59, 1.10	128	0.76	0.57, 1.02	59	0. 80	0.41, 1.06	35	1.09	0.65, 1.82
T3: >121.7	53	**0.55**	**0.38, 0.80**	100	0.84	0.61, 1.15	30	0. 66	0.54, 1.19	28	1.26	0.73, 2.18
*P* for trend		**<0.01**			0.18			0.07			0.41	

*CI, confidence interval; *n*_ca_, number of exposed cases; (ref.), reference category*.

*^a^Tertiles were based on the frequency distribution of energy-adjusted nutrient intakes among controls (men and women combined)*.

*^b^Adjusted for age (continuous), sex (male or female), respondent status (self or proxy), ethnic background (French ancestry, English/Irish/Scottish ancestry, other), education (primary, secondary, post-secondary), daily energy intake (continuous), body mass index (continuous), ever smoked (yes or no), natural log of cigarette-years (continuous), and years since quitting smoking (continuous)*.

*^c^Tests for trend were carried out by modeling nutrient intake tertiles as continuous variables*.

*Bold font indicates P < 0.05*.

### Associations for Selected Fruit and Vegetable Groups

To facilitate comparisons with other studies and describe the relevant food sources in our population, we also evaluated whether specific fruit and vegetable groups high in carotenoids and vitamin C were associated with lung cancer risk. We observed that medium and high weekly intakes of fruits, vegetables, cruciferous vegetables, and citrus fruits, as well as medium intake levels of tomato products and high intakes of carrot products were inversely related to lung cancer risk (Table S3 in Supplementary Material).

## Discussion

In our study, high dietary intakes of β-carotene, α-carotene, β-cryptoxanthin, lycopene, and vitamin C were associated with a decreased risk of lung cancer in main effects analyses. Findings for β-carotene concord with those of a recent meta-analysis in which a pooled relative risk of 0.77 (95% CI: 0.68–0.87) was reported (36). Our results also give credence to a protective role of several other dietary carotenoids on lung cancer and for which evidence was judged to be too limited to draw firm conclusions by the WCRF (7). We found that high intakes of lycopene, a carotenoid which is derived from food sources that tend to differ from those of other carotenoids and vitamin C, lowered the risk of lung cancer. To our knowledge, only one previous study also reported such an association (27). We also found an inverse association between high intakes of vitamin C and lung cancer risk, a finding in line with two studies (33, 35) but at odds with another (31). We did not observe that high intakes of lutein/zeaxanthin significantly lowered the risk of lung cancer, a finding that contrasts with several observational studies (12, 15, 23, 25, 26). A defining characteristic of these studies is that, unlike ours, most were conducted in men exclusively. Nonetheless, there was no indication of effect modification by sex in our study.

Our study is one of the few to have investigated the role of individual antioxidants in separate strata of smoking intensity (20, 22, 27, 31), and the first to simultaneously control for lifetime intensity and time since quitting smoking, two important dimensions of smoking history for lung cancer (48). Although the interactions between micronutrients and smoking intensity were not statistically significant, stratification by cumulative smoking intensity is an improvement over previous studies in which analyses were stratified by smoking status only (i.e., never/former/current smoking) (49), since the former approach is likely to result in more homogenous groups of smokers than the latter. We found that high intakes of β-carotene, α-carotene, β-cryptoxanthin, and lycopene were associated with a reduction in lung cancer risk among male heavy smokers, while vitamin C reduced the risk of lung cancer among female heavy smokers. This suggests that heavy intensity smokers, who suffer from high oxidative stress due to smoking and are at a disproportionate risk of developing lung cancer, can benefit from high intakes of selected antioxidants. Our findings are in line with a number of previous studies. In one study, the associations between antioxidants and lung cancer were stronger among heavy smokers than among never or light smokers, although there was no significant interaction with smoking (31). In another, an inverse association between serum carotenoid levels and lung cancer death was observed among smokers, but not among never/former smokers (50). Our findings for β-carotene are at odds with those from the CARET (51) and the ATBC (52) randomized controlled trials in which β-carotene supplementation in smokers was associated with an increase in lung cancer incidence. However, these associations disappeared when subsequent analyses considered a longer follow-up post-trial (53, 54). Our results regarding never smoking women concord with previous studies (16, 28), although based on small numbers. Furthermore, our data suggest a preventive effect of vitamin C against lung cancer in women who have never smoked, an association which, to our knowledge, has not been reported previously. We also found that women whose cumulative smoking history ranged from 1 to 850 cigarette-years benefited from intakes in the third tertile level of β-carotene and in the second tertile level of β-cryptoxanthin, lycopene, and vitamin C, findings not reported before.

In analyses stratified by histological subtype, we found similar patterns of results by the three most common tumor subtypes, with selected antioxidants being inversely related to risk. We observed that high intakes of β-carotene, α-carotene, β-cryptoxanthin, lycopene, and vitamin C were associated with a reduced risk of squamous cell carcinoma, while high intakes of β-carotene and α-carotene lowered the risk of adenocarcinoma, and both medium and high intakes of β-cryptoxanthin and lycopene reduced the risk of small cell carcinoma. Our results pertaining to risk of squamous cell carcinoma and β-carotene (36), β-cryptoxanthin (26, 32), and vitamin C intakes (20, 26, 33) are comparable to those observed in earlier studies. We found statistically non-significant associations between high intakes of β-carotene, β-cryptoxanthin, lutein/zeaxanthin, lycopene, and vitamin C and an increased risk of large cell carcinoma. To our knowledge, our study is the first to report on dietary antioxidant intakes and risk of large cell carcinoma, a rarer histological subtype (2).

In addition to their antioxidant activity, carotenoids and vitamin C may act to prevent lung carcinogenesis through different mechanisms. For instance, the pro-vitamin A carotenoids such as β-carotene, α-carotene, and β-cryptoxanthin can be metabolized into vitamin A and play a role in cell differentiation (9). β-carotene, β-cryptoxanthin, lutein, zeaxanthin, and lycopene may also inhibit cancerous cell proliferation and contribute to cell-to-cell communication (55–58). In addition, β-carotene and vitamin C have been shown to stimulate apoptosis (57, 59), while α-carotene is thought to inhibit the promotion of carcinogenesis (55, 60). It is therefore conceivable that exposure to carotenoids and vitamin C could play a role in preventing carcinogenesis at different points during the lifetime, which makes them prime candidates for lung cancer prevention.

Fruits and vegetables containing carotenoids and vitamin C are also rich in other nutrients and phytochemicals which could be responsible for their observed protective role against lung cancer. For example, dithiolthiones and isothiocyanates found in cruciferous vegetables and vegetables from the *Allium* genus increase the activity of enzymes involved in carcinogen detoxification (7, 61, 62). Selenium, an essential element, acts as a co-factor for glutathione peroxidase, an enzyme known to protect membranes from oxidation and which also plays a role in carcinogen detoxification (63, 64). Flavonoids are also found in fruits and vegetables, and in our study population, we previously observed a reduced lung cancer risk with intake of some flavonoid subclasses (65). Our results regarding selected groups of fruits and vegetables high in a diversity of chemopreventive micronutrients support their protective role against lung cancer. This is somewhat at odds with results from a recent case–control study conducted in Spain, which found no statistically significant reduction in lung cancer risk associated with consumption of selected fruit and vegetable groups (66) but concords with findings from large meta-analyses (7, 8, 49, 67). One should therefore keep in mind that although dietary intake of carotenoids and vitamin C may help prevent lung cancer, they could also be surrogates for other protective compounds found in selected fruit and vegetables.

Important strengths of our study include the large sample size which allowed for stratification by smoking intensity, for studying specific sources of antioxidants, for examining the impact of diet on different tumor histological subtypes, and for comprehensive control of potential confounding by smoking. Good coverage of the case population in the area and the use of incident, histologically confirmed primary lung cancer cases are also key strengths of our study. Advantages specific to our FFQ are that it contained an extensive food list and was specifically designed to cover major carotenoid and vitamin C sources in the study population. We also used updated local nutrient databases to carry out the conversion from food to nutrient intake.

This study also has some limitations. It would have been of interest to study associations in light of disease aggressiveness, but this information was not available. While the case and control participation rates were high, it is still possible that participating controls were more health conscious than the general population, thus biasing risk estimates away from the null. As well, proxy respondents provided answers for almost 40% of lung cancer cases which could have resulted in measurement error. However, sensitivity analyses indicated that associations between antioxidants and lung cancer did not differ considerably whether we considered self-respondents only or proxy respondents as well. Unmeasured changes in dietary patterns over time are another possible source of bias, although diet has been shown to be relatively stable over time (44). The study was presented to potential and actual participants as a study on environmental factors and the collection of food intake data was one of many themes covered during the interview. These precautions should have reduced the risk that subjects would self-select into the study or report intakes based on their perception of the role of antioxidants on their health status. Finally, although the diet questionnaire focused on the period 2 years prior to diagnosis, cases might have been influenced by their illness when reporting their dietary habits.

Measurement error in dietary intakes inevitably occurred in the study. The FFQ used here was adapted from widely used validated questionnaires (39, 44), which served to develop other FFQ validated and used in various Canadian and Québec populations (38, 68, 69). However, it was not comprehensively tested for reliability and validity in the present study population. Measurement error also likely stemmed from the grouping of more than one food into a single statement, from the lack of information on the exact quantities of foods consumed and on whether foods were consumed cooked or not, and from the relatively broad categories from which subjects could choose to report their intake frequency. We elected to assign mid-point values to each closed category; true intake values might have been close or far from these. Nutrient values attributed to each statement represented the average nutrient content of a food item selected from the statement and under a certain form such as raw or cooked, a choice based on Canadian and Québec food consumption statistics (70, 71). While each of these limitations may have contributed to exposure misclassification, it was likely non-differential with respect to case–control status, thereby attenuating associations. Non-differential misclassification of an exposure with more than two categories may theoretically lead to bias away from the null, but this requires misclassification between two non-adjacent categories, an extreme situation that is unlikely to be present in our data (72). Furthermore, it is highly unlikely that a trend would be reversed by such non-differential misclassification (73). The possibility also remains that the dietary data collected with the FFQ better reflected more recent intakes than remote ones. It could be hypothesized that this latter exposure would be more etiologically relevant with respect to lung cancer development which occurs over many years. Nonetheless, antioxidants are suspected to play a protective role at different times in carcinogenesis. Indeed, antioxidants can inhibit both initiation and promotion stages of tumorigenesis by protecting cells against oxidative damage (74, 75). Therefore, despite the fact that our FFQ assessed more recent antioxidant intakes, it remains a useful measurement tool to capture an effect at the promotion stage.

Finally, although careful attention was given to the control for smoking by using an algorithm shown to be superior to many others in this specific study population (45), residual confounding due to smoking cannot be excluded. Such confounding, if present, could have produced bias toward the null in the estimated associations between nutrient intake and lung cancer risk. However, such a bias could not explain the associations, albeit non-significant ones, observed among the stratum of non-smoking women. We also tested a range of other potential confounding variables such as income, lifetime alcohol intake, and exposure to asbestos, which ultimately were not included in our models because of a lack of effect on the associations.

Overall, our findings contribute both new knowledge and confirmatory data to the as yet inconclusive body of evidence on the hypothesized associations between high intakes of individual carotenoids and vitamin C, and lung cancer risk. They suggest a protective effect of selected antioxidants against lung cancer in general, for different smoking intensity groups and particularly heavy smokers, and for the squamous cell, adenocarcinoma, and small cell histological tumor subtypes. Even though smoking remains the strongest predictor of lung cancer risk, it appears desirable, in light of these findings, to further promote consumption of fruits and vegetables rich in carotenoids and vitamin C to reduce the lung cancer burden among both smokers and non-smokers.

## Ethics Statement

This study was carried out in accordance with the recommendations of McGill University’s Institutional Review Board, the Ethics Committee of the Research Center of Hôtel-Dieu de Montréal and of all individual ethics committees listed in the Material and Methods section, with written informed consent from all subjects. All subjects gave written informed consent in accordance with the Declaration of Helsinki. The protocol was approved by the McGill University’s institutional Review Board (A12-E06-99) and the Ethics Committee of the Research Center of Hôtel-Dieu de Montréal (HDM-941213-18).

## Author Contributions

This manuscript was at the core of MS’s Master’s dissertation. MS conceptualized the research question and was responsible for reviewing the literature, cleaning the data, conducting the analyses, interpreting the results, and writing the manuscript. M-EP and M-CR were MS’s dissertation supervisors and provided overall guidance at all stages of the project and manuscript preparation. AK provided constructive feedback on the manuscript from its first version to the final submitted one. JS and M-EP were co-principal investigators of the original study, provided access to the data, and contributed invaluable feedback as part of manuscript revisions.

## Conflict of Interest Statement

The authors declare that the research was conducted in the absence of any commercial or financial relationships that could be construed as a potential conflict of interest.
