# Supplementary material for: Inverse Association between Dietary Intake of Selected Carotenoids and Vitamin C and Risk of Lung Cancer
Source: Front Oncol. 2017 Feb 28;7:23. doi: 10.3389/fonc.2017.00023 (PMC5328985; doi:10.3389/fonc.2017.00023)
Supplement: Supplementary file 1 [file Data_Sheet_1.DOCX]

Supplementary Table 1. Micronutrient sources

| *β*-carotene | apple, orange, strawberry, cantaloupe, watermelon, apricot, peach, dried apricot, pumpkin pie, tomato, tomato sauce, broccoli, carrot, mixed vegetables, cabbage, iceberg lettuce, spinach, winter squash, other vegetables (green beans, corn, green peas), vegetable soup, orange juice, tomato and vegetable juice |
| --- | --- |
| *α*-carotene | orange, cantaloupe, apricot, pumpkin pie, tomato, tomato sauce, carrot, mixed vegetables, cabbage, iceberg lettuce, winter squash, other vegetables (green beans, corn, green peas), vegetable soup, tomato and vegetable juice |
| *β*-cryptoxanthin | apple, orange, cantaloupe, watermelon, apricot, peach, pumpkin pie, carrot, other vegetables (green beans, corn, green peas), vegetable soup |
| Lutein/zeaxanthin | apple, orange, strawberry, cantaloupe, watermelon, apricot, peach, pumpkin pie, tomato, broccoli, carrot, mixed vegetables, cabbage, iceberg lettuce, spinach, winter squash, other vegetables (green beans, corn, green peas), vegetable soup, apple juice, tomato and vegetable juice |
| Lycopene | melon, tomato, tomato sauce, vegetable soup, tomato and vegetable juice |
| Vitamin C | apple, orange, strawberry, cantaloupe, watermelon, apricot, peach, dried apricot, pumpkin pie, tomato, tomato sauce, broccoli, carrot, mixed vegetables, cabbage, iceberg lettuce, spinach, winter squash, other vegetables (green beans, corn, green peas), vegetable soup, orange juice, apple juice, tomato and vegetable juice |

Supplementary Table 2. Bivariate correlations between daily micronutrient intakes

|  | *α*-carotene | *β*-cryptoxanthin | Lutein/zeaxanthin | Lycopene | Vitamin C |
| --- | --- | --- | --- | --- | --- |
| *β*-carotene | 0.939 | 0.759 | 0.778 | 0.499 | 0.678 |
| *α*-carotene |  | 0.725 | 0.532 | 0.457 | 0.570 |
| *β*-cryptoxanthin |  |  | 0.519 | 0.364 | 0.824 |
| Lutein/zeaxanthin |  |  |  | 0.340 | 0.574 |
| Lycopene |  |  |  |  | 0.381 |

Supplementary Table 3. Adjusted^a^ ORs and tests for trend for lung cancer risk according to weekly intake of fruit and vegetable groups, Montreal, QC, Canada, 1996-2002

| Portions/week^b,c^ | n_ca_ | OR | 95% CI |
| --- | --- | --- | --- |
| Fruit |  |  |  |
| T1: <= 13.0 | 659 | (ref.) |  |
| T2: 13.5 – 21.1 | 245 | **0.66**^d^ | **0.52, 0.86** |
| T3 : >= 22 | 201 | **0.59** | **0.44, 0.79** |
| *P* for trend |  | **<0.001** |  |
| Vegetables |  |  |  |
| T1: <= 15.0 | 611 | (ref.) |  |
| T2: 15.5 – 22.5 | 298 | **0.74** | **0.58, 0.94** |
| T3 : >= 23.5 | 196 | **0.58** | **0.43, 0.78** |
| *P* for trend |  | **<0.001** |  |
| Cruciferous vegetables |  |  |  |
| T1: <= 2.0 | 604 | (ref.) |  |
| T2: 2.5 – 4.5 | 255 | **0.76** | **0.59, 0.98** |
| T3 : >= 5.0 | 246 | **0.66** | **0.50, 0.85** |
| *P* for trend |  | **<0.001** |  |
| Leafy vegetables |  |  |  |
| T1: <= 2.0 | 608 | (ref.) |  |
| T2: 2.5 – 4.0 | 258 | 1.00 | 0.78, 1.30 |
| T3 : >= 4.5 | 239 | 0.80 | 0.62, 1.04 |
| *P* for trend |  | 0.11 |  |
| Citrus |  |  |  |
| T1: <= 4.0 | 600 | (ref.) |  |
| T2: 4.5 – 7.5 | 283 | **0.77** | **0.60, 0.98** |
| T3 : >= 8.0 | 222 | **0.68** | **0.52, 0.90** |
| *P* for trend |  | **0.01** |  |
| Tomato products |  |  |  |
| T1: <= 3.0 | 471 | (ref.) |  |
| T2: 3.5 – 5.5 | 298 | **0.66** | **0.51, 0.84** |
| T3 : >= 6.0 | 336 | 0.81 | 0.63, 1.04 |
| *P* for trend |  | 0.09 |  |
| Carrots |  |  |  |
| T1: <= 2.0 | 640 | (ref.) |  |
| T2: 2.5 – 4.0 | 288 | 0.88 | 0.69, 1.11 |
| T3 : >= 4.5 | 177 | **0.66** | **0.50, 0.88** |
| *P* for trend |  | **0.01** |  |

CI, confidence interval; n_ca_, number of exposed cases; OR, odds ratio; (ref.), reference category.

^a^Models adjusted for age (continuous), sex (man or woman), respondent status (self or proxy), ethnic background (French ancestry, English/Irish/Scottish ancestry, other), education (primary, secondary, post-secondary), ever smoked (yes or no), natural log of cigarette-years (continuous), years since quitting smoking (continuous), energy intake (continuous), and body mass index (continuous).

^b^Fruit and vegetables included in each category are the following: Fruit: apple, orange, strawberry, cantaloupe, watermelon, apricot, peach, dried apricot, other fruit, orange juice, apple juice; Vegetables: pumpkin pie, tomato, tomato sauce, broccoli, carrot, mixed vegetables, cabbage, iceberg lettuce, spinach, winter squash, other vegetables (green beans, corn, green peas), vegetable soup, tomato and vegetable juice; Cruciferous vegetables: broccoli, cabbage, spinach; Leafy vegetables: iceberg lettuce, spinach; Citrus: orange, orange juice; Tomato products: tomato, tomato sauce, tomato and vegetable juice; Carrots: carrots, mixed vegetables.

^c^Tertiles based on the frequency distribution of intakes among controls (men and women combined).

^d^Bold font indicates *P* < 0.05
